# Bilingual Language Experience Shapes Resting-State Brain Rhythms

**DOI:** 10.1162/nol_a_00014

**Published:** 2020-07-01

**Authors:** Kinsey Bice, Brianna L. Yamasaki, Chantel S. Prat

**Affiliations:** Institute for Learning and Brain Sciences and Department of Psychology, University of Washington; Institute for Learning and Brain Sciences and Department of Psychology, University of Washington; Department of Psychology and Human Development, Vanderbilt University; Institute for Learning and Brain Sciences and Department of Psychology, University of Washington

**Keywords:** bilingualism, neural oscillations, resting-state, EEGs

## Abstract

An increasing body of research has investigated how bilingual language experience changes brain structure and function, including changes to task-free, or “resting-state” brain connectivity. Such findings provide important evidence about how the brain continues to be shaped by different language experiences throughout the lifespan. The neural effects of bilingual language experience can provide evidence about the additional processing demands placed on the linguistic and/or executive systems by dual-language use. While considerable research has used MRI to examine *where* these changes occur, such methods cannot reveal the temporal dynamics of functioning brain networks at rest. The current study used data from task-free EEGS to disentangle how the linguistic and cognitive demands of bilingual language use impact brain functioning. Data analyzed from 106 bilinguals and 91 monolinguals revealed that bilinguals had greater alpha power, and significantly greater and broader coherence in the alpha and beta frequency ranges than monolinguals. Follow-up analyses showed that higher alpha was related to language control: more second-language use, higher native-language proficiency, and earlier age of second-language acquisition. Bilateral beta power was related to native-language proficiency, whereas theta was related to native-language proficiency only in left-hemisphere electrodes. The results contribute to our understanding of how the linguistic and cognitive requirements of dual-language use shape intrinsic brain activity, and what the broader implications for information processing may be.

## INTRODUCTION

Although language is a human universal, variability in language experience has been shown to shape the brain (Li, Legault, & Litcofsky, 2014; Pliatsikas, Moschopoulou, & Saddy, 2015; Wong, Yin, & O’Brien, 2016), and therefore, how the brain processes information (e.g., Onnis & Thiessen, 2013; Yamasaki, Stocco, & Prat, 2018). Bilingualism, or the ability to use two languages proficiently, is a particular class of language experience that characterizes more than half of the world’s population (Grosjean, 2014); yet, the mechanisms by which bilingual individuals fluently use multiple languages remains relatively poorly understood. An increasing number of behavioral (e.g., Bogulski, Bice, & Kroll, 2018; Yamasaki & Prat, 2014; Yamasaki et al., 2018) and neuroscientific (e.g., Abutalebi & Green, 2007; Abutalebi & Green, 2016; Seo & Prat, 2019; Stocco, Yamasaki, Natalenko, & Prat, 2014) studies have suggested that speaking more than one language creates *unique* demands for bilingual individuals. Linguistically, bilingual language use requires maintaining two interconnected but separable language systems (phonological maps, lexicons, grammars, etc.), and managing interference that stems from their coactivation (e.g., Kroll, Bobb, & Wodniecka, 2006; Kroll, Dussias, Bice, & Perrotti, 2015). Cognitively, bilinguals must monitor the language(s) in their environments and dynamically select the intended target language, which places additional demands on nonlinguistic executive functions (e.g., Bialystok, Craik, & Luk, 2012; Green & Abutalebi, 2013; Stocco & Prat, 2014). Given these additional linguistic and cognitive demands, it is perhaps unsurprising that bilingual language experience produces both structural and functional changes in the brain (Li et al., 2014; Pliatsikas et al., 2015; Wong et al., 2016).

In an effort to gain a better understanding of the mechanisms underpinning bilingual language control, an increasing amount of research has examined the neural impact of various facets of bilingual language experience (e.g., DeLuca, Rothman, Bialystok, & Pliatsikas, 2019; Green & Abutalebi, 2013; Gullifer et al., 2018; Legault, Fang, Lan, & Li, 2019). As a whole, this body of research has demonstrated that the unique demands of bilingualism, and the different forms that bilingualism may take under different circumstances, give rise to measurable changes throughout the brain. Much of this research has given particular attention to general cognitive control regions such as the anterior cingulate cortex (Abutalebi & Green, 2007; Abutalebi et al., 2011) and the basal ganglia nuclei (Abutalebi et al., 2013; Hervais-Adelman, Moser-Mercer, & Golestani, 2011; Seo, Stocco, & Prat, 2018). Different facets of bilingual language experience, such as a bilingual’s current balance of dual-language use and relative proficiency in each language, have been shown to drive differences in these cognitive control regions (Abutalebi et al., 2013; Gullifer et al., 2018). These findings are consistent with prominent theories such as the adaptive control hypothesis (Green & Abutalebi, 2013) and with studies measuring language control online in bilinguals (Guo, Liu, Misra, & Kroll, 2011; Rossi, Newman, Kroll, & Diaz, 2018; Seo et al., 2018; Seo & Prat, 2019), which also implicate general cognitive control mechanisms in bilingual language use.

Given the research showing that bilinguals rely on domain-general cognitive control regions to accomplish dual-language use, a number of experiments have considered whether the effects of dual-language use can be observed even in the absence of tasks specifically requiring language. For example, several studies have shown that the way bilinguals use and control language influences their neural and behavioral responses on nonlinguistic tasks (e.g., Abutalebi et al., 2011; Bialystok, Poarch, Luo, & Craik, 2014; Stocco & Prat, 2014; Yamasaki, Stocco, Liu, & Prat, 2019; Yamasaki et al., 2018). Remarkably, the impacts of dual-language use can even be observed in more static, task-free measures, which suggests that underlying brain function is shifted as a result of bilingualism, as opposed to simply changing how bilinguals perform tasks. Perhaps unsurprisingly, the same regions shown to be recruited during tasks that require bilingual language control (e.g., the basal ganglia nuclei and the anterior cingulate) are also those that most consistently show long-term structural and functional changes in task-free activity. This observation suggests that the mechanisms employed during on-task bilingual language control shape task-free brain activity, ultimately producing changes to structural features and functional connectivity in the brain.

Task-free, or “resting-state,” brain function and connectivity is a measure of the brain’s networks during resting wakefulness, which captures the dynamic and spontaneous activity that is continuously produced by the brain. It is thought to reflect local and long-range coordination of brain regions in the act of maintaining and updating pathways due to recent experiences (Raichle & Snyder, 2007). As such, task-free brain activity has been shown to shift slowly over time across developmental periods from childhood through older adulthood (Anderson & Perone, 2018; Klimesch, 1999) but is otherwise relatively stable (Salinsky, Oken, & Morehead, 1991).

Some have conceptualized intrinsic brain activity as the brain’s “readiness” to predict and integrate new experiences, consistent with a Bayesian perspective of brain functioning (Albert, Robertson, & Miall, 2009; Miall & Robertson, 2006; Raichle & Snyder, 2007; Stevens & Spreng, 2014). That is, infants are born with a set of “priors” that generate predictions about the surrounding world, and as those predictions are violated throughout the course of one’s life, they are adjusted and shaped by consistent experiences to continually provide the best possible prediction for current and future contexts. Therefore, as experiences accumulate over the lifespan, especially repeated experiences like dual-language use, they become ingrained in the patterns of intrinsic brain function. Consistent with this hypothesis, many different types of expertise have been associated with changes in task-free brain activity, including that of jugglers (Gerber et al., 2014), spatial navigators (Maguire et al., 2003), meditators (Brewer et al., 2011), musicians (Luo et al., 2012), and athletes (Babiloni et al., 2010; Di et al., 2012; Muraskin et al., 2016). Single, extreme experiences can also induce temporary shifts to the patterns of brain activity (Rossi, Bice, Kürüm, & Kroll, in preparation; for a review, see Stevens & Spreng, 2014), but experiences that are repeated or protracted, like language use over the lifespan, are more reliably encoded long-term.

Most of the existing research on task-free measures in bilinguals has focused on anatomical measures obtained using fMRI measures, such as *where* structural changes to grey and white matter density can be observed in bilinguals (Klein, Mok, Chen, & Watkins, 2014). Research using functional measures have similarly focused primarily on differences in the spatial distribution, or on the degree of connectivity between anatomically defined regions, in resting-state networks as a function of bilingual language experience (e.g., Berken, Chai, Chen, Gracco, & Klein, 2016; Grady, Luk, Craik, & Bialystok, 2015; Gullifer et al., 2018; Kousaie, Chai, Sander, & Klein, 2017). Changes in these task-free neural indices, which are relatively stable across time, provide hints about how bilingual language experience shapes brain function more broadly. However, these inferences are limited to information about *where* bilingual language experience shapes the brain. Because many of the brain areas and networks implicated in bilingual language control are broadly involved in general executive processes, and because the time course of fMRI data reduces our ability to make inferences about *what* these regions might be contributing to bilingual language control, the current experiment involved a complementary investigation of individual differences in resting-state electroencephalography (EEG).

EEGs and their oscillatory components have a long and rich history of research uncovering how synchronized activity in different frequency ranges communicates, coordinates, and conveys information. Unlike resting-state fMRI measures that can only capture slow-wave fluctuations in brain activity which correspond to a flurry of neural activity, EEGs provide the temporal sensitivity to allow more refined inferences about information processing in the brain. Importantly for research investigating the impact of bilingual language experience on the mind and brain, variation in the frequency of EEG oscillations has been linked to specific *mechanisms* that subserve relevant cognitive processes such as cognitive control (Cavanagh & Frank, 2014; Klimesch, Sauseng, & Hanslmayr, 2007; Sadaghiani & Kleinschmidt, 2016), working memory (Miller, Lundqvist, & Bastos, 2018, and language processing (Bastiaansen & Hagoort, 2006; Giraud & Poeppel, 2012; Weiss & Mueller, 2012). These mechanisms work by coordinating spike timing within and across neural assemblies, by binding together information that unfolds over time and echoing that information to maintain it in mind, and by managing activity from other neural assemblies (Buzsáki, 2006). For example, lower frequencies (e.g., theta around 4–7 Hz) are used for long-distance communication (von Stein & Sarnthein, 2000), even between regions that are only indirectly connected, thus providing an additional window into brain functioning that cannot be revealed through fMRI measures. Higher frequencies (e.g., gamma around 30–60 Hz) are typically associated with more local processing (von Stein & Sarnthein, 2000) and have been described as the neural “letters” that are combined into different “words” through cross-coupling with slower frequencies (Buzsáki & Watson, 2012). Many have argued that oscillations are the “neural syntax” that serve to bind important information together (Buzsáki & Watson, 2012) and that intrinsic oscillations, which are not task-dependent, are critical for understanding dynamic excitatory-inhibitory balances, (interregional) communication, and neural function more broadly.

By using EEGs to examine differences in task-free brain activity, the current study sought to investigate the mechanism(s) that translate online information processing during bilingual language use to changes observed in task-free brain activity. As one of the most prominent features of task-free brain activity, alpha has been studied perhaps the most, or at least the longest, of any of the frequency bands during resting state (Berger, 1929). Alpha activity exhibits large inter-individual variability that is related to a number of cognitive processes, including processing speed (Klimesch, Doppelmayr, Schimke, & Pachinger, 1996), intelligence (e.g., Anokhin & Vogel, 1996; Doppelmayr, Klimesch, Stadler, Pöllhuber, & Heine, 2002), attention (Klimesch, Schimke, & Pfurtscheller, 1993), language processing (Bornkessel, Fiebach, Friederici, & Schlesewsky, 2004), and especially inhibitory control (Klimesch et al., 2007; Strauß, Wöstmann, Obleser, 2014). It is most easily observed in thalamo-cortical circuits (Başar, Schürmann, Başar-Eroglu, & Karakaş, 1997), particularly during periods of disengagement. Alpha can at times appear to behave paradoxically; in general, higher alpha power at rest and lower alpha power on-task index good performance, yet greater increases in alpha power during more difficult tasks also lead to better performance. These findings have been reconciled with an inhibitory account of alpha function (Cooper, Croft, Dominey, Burgess, & Gruzelier, 2003; Jensen & Mazaheri, 2010; Klimesch et al., 2007), such that alpha activity is related to attentional control and inhibition of irrelevant cortical activity. In the absence of a task, cortical activity related to sensory/perceptual processes becomes irrelevant while a person focuses inward, thus alpha power increases to reduce signal and interference from sensory regions, especially over the occipital electrodes. In general, lower alpha on task suggests engagement and widespread functioning, but when task demands increase and require greater focus and control, alpha intervenes as an inhibitory mechanism to focus brain activity on the most relevant regions, thus increasing signal-to-noise and reducing interference from other areas. A wealth of research has reported evidence in favor of the inhibitory account (Scheeringa, Petersson, Kleinschmidt, Jensen, & Bastiaansen, 2012), featuring alpha power, coherence (Klimesch et al., 2007), and phase coupling (Sadaghiani et al., 2012) as mechanisms by which alpha can be used to exert cognitive control.

Under this inhibitory account, alpha is seen as a general mechanism that subserves various cognitive processes that utilize inhibitory control. One such cognitive process is dual-language use, which has been shown to specifically shape brain regions associated with inhibitory control for language (e.g., anterior cingulate cortex, basal ganglia). While monolingual speakers must resolve competition between alternatives within their language to select words and structures to use, bilinguals additionally experience the coactivation of alternatives in both of their languages (Kroll, Bobb, & Hoshino, 2014; Kroll et al., 2006). The constant requirement to monitor language contexts, maintain target language goals, and resolve cross-linguistic conflict changes how bilinguals engage and coordinate cognitive control with their language processes to accomplish dual-language use. Bilingual language use requires the linguistic system to rely more heavily on domain-general cognitive control resources that are recruited to resolve the extra layer of cross-language competition (e.g., Li et al., 2014; Stocco et al., 2014; Yamasaki & Prat, 2020). Many studies report that bilinguals engage subcortical structures (e.g., caudate, putamen, and anterior cingulate cortex) to route and resolve conflict and that greater bilingual skill is associated with less reliance on frontal structures for conflict resolution but instead shifts to subcortical or posterior regions (e.g., Grundy, Anderson, & Bialystok, 2017). Given its mechanistic role in inhibitory control, we hypothesized that alpha would be a critical frequency band impacted by the demands of dual-language knowledge or use.

Similarly, the beta frequency band is one of the primary frequencies engaged in cortico-basal ganglia loops (e.g., Brittain, Sharott, & Brown, 2014; Stein & Bar-Gad, 2013), which play an important role in language processing (e.g., Booth, Wood, Lu, Houk, & Bitan, 2007; Kotz, Schwartze, & Schmidt-Kassow, 2009), especially dual-language use (e.g., Buchweitz & Prat, 2013; Seo et al., 2018; Stocco & Prat, 2014). Beta mechanisms have been proposed to serve a maintenance role (Engel & Fries, 2010), particularly for maintenance in working memory (Miller, Lundqvist, & Bastos 2018). Newer models of the role of neural oscillations in working memory have proposed that beta and gamma work jointly to negotiate top-down (beta) and bottom-up (gamma) maintenance, control, and shifting in working memory (Miller et al., 2018). The beta frequency plays an important role in language because it helps to temporally bind and integrate multisensory linguistic information unfolding over time, both to process incoming information and to maintain the past context and transitional or probability constraints from previous time windows. This role puts the beta frequency at the interface between language and working memory, maintenance, and filtering (Miller et al., 2018; Weiss & Mueller, 2012). For example, during language comprehension beta power decreases upon encountering an unexpected word in sentences that have strong semantic context, in line with its role in maintaining the current trajectory and predicting upcoming information (Lewis & Bastiaansen, 2015; Lewis, Wang, & Bastiaansen, 2015; Wang et al., 2012).

Importantly, the majority of the research on beta and language or other cognitive processes comes from on-task performance, with relatively little examining how beta at rest is linked to later performance on task (except see Prat, Yamasaki, Kluender, & Stocco, 2016; Prat, Yamasaki, & Peterson, 2019). The few studies that have examined beta power at rest have found that higher beta power over the right temporal electrodes significantly predicts individual differences in language learning rates at the initial stages of learning (Prat et al., 2016), and that bilateral beta coherence over frontal electrodes is related to a language learner’s willingness to speak and use the language (Prat et al., 2019).

Because of its role in language, contextual sensitivity, and maintenance, we hypothesized that activity in the beta frequency band would be related to features of bilingual language experience. Active bilingual language use imposes qualitatively and quantitatively different demands on the cognitive and linguistic systems through the requirement to maintain multiple sound inventories, partially overlapping semantic and lexical systems, and awareness of appropriate grammatical structures. Beta is found in basal ganglia–frontal cortex circuits, which are important for tracking one’s ongoing context and allowing contextually appropriate signals through to the frontal cortex for further processing and action. The statistics of the surrounding environment for active bilinguals are much more varied and complex than the statistics needed for a monolingual to comprehend and use a single language. One possible reason for basal ganglia differences, and hypothesized beta differences, in bilinguals is to help with the additional demands of sampling and tracking statistics of the surrounding context, which assists with predicting upcoming events or information. These processes in addition to the other demands on the bilingual’s linguistic system (e.g., Gollan, Montoya, Cera, & Sandoval, 2008; Ivanova & Costa, 2008; Libben & Titone, 2009) likely change how bilinguals recruit and use beta mechanisms.

Few previous studies have examined task-free oscillatory brain networks in bilinguals. One study used MEG to compare functional connectivity between healthy older monolingual and bilingual adults (de Frutos-Lucas et al., 2019). They found that bilinguals had greater functional connectivity between posterior regions than monolinguals in three frequency ranges, but monolinguals did not exhibit higher functional connectivity than bilinguals between regions in any of the frequency ranges examined. Specifically, bilingual older adults had higher connectivity between bilateral occipital regions in the theta (4–8 Hz) and high beta (20–30 Hz) bands, between left occipital and left parietal regions in the low beta (12–20 Hz) and high beta bands, and within left posterior regions in the theta band. Given that the anterior regions of the brain tend to deteriorate more/faster during the aging process, they interpreted their results showing greater preservation of posterior brain networks within the bilingual older adults as a potential source of cognitive reserve. Their findings are in line with other studies that have shown that bilingualism imposes a shift from reliance on frontal control networks to subcortical and/or posterior brain regions (see Grundy et al., 2017). Essentially, as a result of the shift in processing from anterior to posterior regions, bilinguals may be better able to cope with the anterior degradation found in normal, healthy aging as well as pathological aging, thus producing cognitive reserve.

Brain activity measured with EEGs can be characterized by either measures of power or coherence. Measures of power capture increased or decreased synchronicity of the generating neurons measured at a single electrode, which could be due to a larger network of neurons and/or to a smaller group of neurons that are firing synchronously. Measures of coherence are more akin to indices of functional connectivity, in that they capture longer range relations between activity in different brain regions or groups of generating neurons measured across electrodes. Given the similarities between coherence and functional connectivity, the results from de Frutos-Lucas et al. (2019) suggest that we should expect differences in coherence between bilinguals and monolinguals, but less is known about whether to expect differences in power.

### The Current Study

The current study aimed to compare intrinsic brain function, measured using EEGs, between large samples of young adult monolinguals and bilinguals. Bilingual language use has been shown to shape cognitive processes involved in language and inhibitory control, whose repeated engagement produces structural and functional changes to brain regions associated with cognitive control. Alpha and beta frequencies are both general mechanisms involved in top-down control. Alpha can be found across cortical regions and is a powerful and general mechanism for inhibiting cortical activation, and beta is prevalent in the basal ganglia and its circuits, which have been shown to differ in bilingual speakers. Given previous research on the cognitive functions associated with the alpha and beta frequencies, we hypothesized that bilinguals and monolinguals would differ in alpha and beta activity in task-free states, and that individual differences in alpha and beta would be related to aspects of language experience (e.g., proficiency, age of acquisition, proportion of current dual-language use) and/or cognitive control (measured via a Simon task). However, given that this study represents one of the first studies to systematically report task-free EEG differences between bilinguals and monolinguals, we report a full set of analyses including all frequency bands, not restricted to the frequencies of interest (alpha and beta).

## METHODS

### Participants

The study included data from 91 monolingual speakers (61 female) and 106 bilingual speakers (81 female). Descriptive statistics for various demographic and linguistic variables across groups can be found in Table 1. Participants were aged 18–35, with no significant difference in age between the two groups, *t*(195) = 1.63, *p* > 0.10. All participants had a minimum education level equivalent to a high school GED (∼12 years of education), up to a Ph.D. (∼24 years of education); the two groups did not differ significantly in the number of years of education they reported, *t*(175) = 0.73, *p* > 0.10. To be included as a bilingual in the study, bilinguals must have self-identified as bilingual and have self-reported their average proficiency in the ability to speak, read, and understand a second-acquired language (L2) as no lower than 5 on a scale from 1 (*no skills*) to 10 (*extremely proficient*). Among the bilinguals, 65 considered a language other than English to be both their native and dominant language, 37 reported having a language other than English as their native language but currently considered English to be their dominant language, 1 whose native language was English but English was currently not their dominant language, and 3 whose native language and dominant language were English but had also acquired high proficiency in another language. The majority of the bilinguals were early but not simultaneous bilinguals; only 15 acquired both languages before the age of 5, but 66 acquired their L2 between the ages of 5 and 10.

**Table 1.** Demographic and linguistic descriptive statistics for bilingual and monolingual participants

	Bilinguals: L1	Bilinguals: L2	Monolinguals: L1	Monolinguals: L2
Language(s)	Mandarin = 56 Korean = 14 Spanish = 8 Cantonese = 7 Japanese = 6 Vietnamese = 6 English = 4 Bengali = 1 French = 1 Gujarati = 1 Indonesian = 1 Latvian = 1	English = 102 Spanish = 4	English = 91	Spanish = 10 French = 2 Portuguese = 1
Self-Reported^a^ Speaking	9.11 (1.13) Range: 6–10	7.99 (1.25) Range: 5–10	9.85 (0.41) Range: 8–10	2.31 (1.18) Range: 0–4
Self-Reported^a^ Understanding	9.22 (1.27) Range: 3–10	8.25 (1.27) Range: 5–10	9.88 (0.36) Range: 9–10	2.85 (0.8) Range: 2–4
Self-Reported^a^ Reading	8.58 (2.26) Range: 0–10*	7.98 (1.36) Range: 5–10	9.80 (0.48) Range: 8–10	2.50 (1.41) Range: 1–5
Age of Acquisition^b^		7.26 (2.97) Range: 0–18		13.23 (2.42) Range: 8–17
Proportion of Use: Speaking^c^	42.98 (24.10) Range: 0–100	54.74 (23.77) Range: 0–100	99.76 (1.07) Range: 92–100	1.50 (2.50) Range: 0–8
Proportion of Use: Listening^c^	36.65 (21.27) Range: 0–100	60.43 (21.37) Range: 0–100	99.82 (0.66) Range: 95–100	1.00 (1.41) Range: 0–5
Proportion of Use: Reading^c^	28.58 (25.32) Range: 0–100	69.84 (25.14) Range: 0–100	99.85 (1.11) Range: 90–100	0.75 (2.30) Range: 0–8
Simon Effect RTs^d^ (ms)	80.19 (46.5) Range: 11–306		63.71 (38.19) Range: −30–178	
Simon Effect ACCs^d^	−0.01 (0.14) Range: −0.47–0.53		0.08 (0.12) Range: −0.4–0.42	

^a^ Self-reported measures were taken from a modified version of the LEAP-Q using a scale from 1 (*no skills*) to 10 (*extremely proficient*).

^b^ Age of acquisition was a free-report estimate in which participants indicated at what age they began acquiring the specified language.

^c^ Proportion of use was a free-report estimate in which participants indicated how much time, on average, they spent speaking or listening to each of their language(s), such that all estimates added up to 100%; only up to two languages were included in analyses.

^d^ Simon effect was calculated for response times (RTs) by subtracting the average correct congruent RTs from incongruent RTs, and for accuracy rates (ACCs) by subtracting the incongruent ACCs from the congruent ACCs.

* Low self-reported reading estimates are from heritage speakers born in the USA with a home language that uses a non-Roman script (Cantonese, Gujarati).

To be considered monolingual, participants must have considered themselves monolingual (or “functionally monolingual”), as well as have reported their average proficiency in speaking, reading, and understanding any language other than English as 4 or less on a scale from 1 (*no skills*) to 10 (*extremely proficient*). Thirteen monolinguals reported some knowledge of another language through foreign language classes or some other experience, but given the criteria for being considered monolingual, their self-ratings in their L2 were significantly lower than bilinguals’ ratings in their second language. Monolinguals with L2 experience reported lower ability in speaking (*M* = 2.31, *SD* = 1.18), understanding (*M* = 2.85, *SD* = 0.80), and reading (*M* = 2.85, *SD* = 1.41) than bilinguals, *M* = 7.99, *SD* = 1.25, *t*(117) = 15.58, *p* < 0.001; *M* = 8.25, *SD* = 1.27, *t*(117) = 15.00, *p* < 0.001; and *M* = 7.98, *SD* = 1.37, *t*(117) = 12.73, *p* < 0.001, respectively. Furthermore, bilinguals acquired their L2 significantly earlier than monolinguals, *t*(19.02) = 8.64, *p* < 0.001. On average, bilinguals began learning their L2 at age 7.26 years (*SD* = 2.97), and monolinguals who reported studying a L2 began learning the language at age 13.23 (*SD* = 2.42).

In their native or first-acquired language (L1), bilinguals reported lower self-ratings of their ability to speak (*M* = 9.11, *SD* = 1.13), understand (*M* = 9.23, *SD* = 1.27), and read (*M* = 8.58, *SD* = 2.26) than monolinguals, *M* = 9.86, *SD* = 0.41, *t*(195) = 5.99, *p* < 0.001; *M* = 9.88, *SD* = 0.36, *t*(195) = 4.37, *p* < 0.001; and *M* = 9.80, *SD* = 0.48, *t*(195) = 5.06, *p* < 0.001, respectively. For bilinguals, there was a negative correlation between averaged self-rated proficiency in their L1 and L2, *r*(100) = −.35, *p* < 0.001.

### Materials and Procedure

All participants volunteered to participate in the study and provided informed consent, and all procedures were approved by the Human Subjects Division Institutional Review Board at the University of Washington. Participants included in the current study were recruited for a variety of different studies in the lab, including studies on L2 learning, L2 reading skill, and neurofeedback training (Prat et al., 2016; Prat et al., 2019). During the EEG recording session, participants were fit with the Emotiv™ EEG headset. Five-minutes of task-free data were collected from each participant while they were seated in a dark, quiet room with their eyes closed. All participants additionally completed a common set of questionnaires and computerized tasks across studies: an adapted version of the Language Experience and Proficiency Questionnaire (LEAP-Q; Marian, Blumfeld, & Kaushanskaya, 2007), a demographics questionnaire, and the Simon task (Stocco et al., 2017). In the Simon task, participants were shown a black square or circle on a white background and had to respond to each shape with a different hand (counterbalanced) by pressing the letter “Q” with the left hand or the number 7 on the numeric keypad with the right hand. The trials were preceded by 800 ms fixation and 250 ms blank screen, after which the stimulus appeared on the screen until a response was made or up to a maximum of 3,000 ms. Seventy-five percent of the trials were congruent (i.e., the shape associated with the left-handed response appeared on the left side of the screen and vice versa), and the remaining 25% of the trials were incongruent (i.e., the shape associated with the left-handed response appeared on the right side of the screen and vice versa). A total of 60 trials were presented in random order.

### EEG Acquisition and Processing

EEGs were collected from 14 scalp electrodes (AF3, AF4, F3, F4, F7, F8, FC5, FC6, T7, T8, P7, P8, O1, O2) and two reference electrodes (CMS, DRL) at a sampling rate of 128 Hz using the Emotiv headsets.

EEGs were processed offline using R programming and packages (Hothorn, Hornik, van de Wiel, & Zeileis, 2008; R Core Team, 2017; Wickham, 2011). Each participant’s EEG data were split into 2 s windows with 50% overlap between segments. Any segment containing artifacts, defined by segments with deviations in the waveform more than 3 standard deviations above or below the channel’s average activity, or segments in which the Emotiv headset detected eye blinks or large gyroscope movements, were excluded from further processing. Using a fast Fourier transform algorithm, each segment was decomposed into the frequency domain and then averaged together. The log power at each frequency (from 0.5 to 40 Hz in increments of 0.5 Hz) for each participant and electrode and the correlated activity between two electrodes (i.e., coherence) at each frequency step was used in further analyses.

#### Individualized frequency bands

Because of the continuous nature of the frequency spectrum, most studies bin frequencies into different bands (delta: ∼0–4 Hz, theta: ∼4–8 Hz, alpha: ∼8–12 Hz, beta: ∼12–30 Hz, and gamma: ∼30+ Hz), which are then used for further analyses. The boundaries of the frequency bands, however, are somewhat arbitrary, and vary across studies. Recently, researchers have advocated for individualized frequency bands that are anchored to each person’s individual alpha frequency (IAF; Klimesch, 1997). The IAF is the frequency at which an individual’s synchronous brain activity peaks, which tends to be between 8–14 Hz and is predominantly found over posterior electrodes, when measured during eyes-closed resting state. Once each individual’s peak alpha frequency is determined, the frequency bands can be drawn with respect to the IAF (e.g., for an individual whose IAF = 10 Hz, their alpha frequency band would be from 8–12 Hz, as compared with an individual whose IAF = 12 Hz, whose band would be from 10–14 Hz). Several studies have found that the IAF is related to measures of intelligence (Doppelmayr et al., 2002) and processing speed (Klimesch et al., 2007) and that experimentally increasing a person’s IAF using transcranial alternating current stimulation at the person’s IAF + 1 Hz or through neurofeedback training improves cognitive performance (Zaehle, Rach, & Herrmann, 2010; Zoefel, Huster, & Herrmann, 2011). Given the known variability in IAF (Haegens, Cousijn, Wallis, Harrison, & Nobre, 2014), the current study defined frequency bands with respect to each participant’s IAF using a modified version of the individual alpha frequency fixed bandwidth methods described by Doppelmayr, Klimesch, Pachinger, and Ripper (1998). These individualized frequency bands were used for all subsequent analyses, which allowed us to investigate group differences in the IAF as well as the brain’s peak power at the IAF in different electrode sites.

The process of identifying the IAF for each participant involved multiple exclusionary steps prior to peak detection. Broadly speaking, the exclusionary steps involved removing channels with bad data, removing channels that lacked an alpha peak, and removing participants who had fewer than 7 “good” channels (half the channels) remaining due to low reliability of the IAF estimation with fewer than 7 channels. First, any channels with unusually high or low activity across the frequency spectrum that might bias measurements of power in each frequency band were excluded. To achieve this, for each participant, log power within 1–40 Hz on the frequency spectrum across all of the channels was averaged, and then any channel whose average log power was more than 2.5 standard deviations above or below the all channel average was excluded. This resulted in the exclusion of 60 channels (2.1% of the data), with never more than 1 channel per participant excluded (channels excluded: AF4 = 2, F3 = 1, F4 = 1, F7 = 4, F8 = 10, FC6 = 2, O1 = 3, O2 = 3, P7 = 13, P8 = 4, T7 = 13, T8 = 4). The channels identified with high or low activity were excluded from both the IAF and the individualized frequency band calculations, as well as the subsequent calculation of average power and coherence within each individualized frequency band.

To estimate each individual’s IAF, any channels that lacked an alpha peak were additionally excluded. Alpha peaks were defined as a minimum increase of 0.2 log(mV^2) within a liberal alpha frequency range (7.5–14 Hz). A total of 245 channels were excluded for lacking an alpha peak (8.9% of the data). However, these channels were only excluded from the IAF estimation; after each individual’s IAF was estimated, these channels were once again included in the calculation of averaged power and coherence within each individualized frequency band.

Finally, in order to ensure a more stable estimate of IAF, any participants who had fewer than 7 channels (half) remaining after the first two exclusionary steps were additionally excluded from IAF, power, and coherence calculations. Seven participants (6 monolinguals, 1 bilingual) were excluded for having fewer than 7 channels remaining. Importantly, all of these excluded participants had at least 7 channels removed for lacking an alpha peak, rather than for having a bad spectrum, so the primary reason for exclusion was not due to poor data quality. Individuals for whom we could not estimate an IAF were excluded from all further analyses.

For each participant that met the inclusionary criteria, the IAF was determined by averaging the spectra across all good channels and then identifying the alpha peak within a liberal alpha frequency range (7.5–14 Hz). This process resulted in a single IAF value for each participant. Frequency bands were defined with respect to each participant’s IAF, with IAF representing 0 on a relative frequency spectrum. Delta was defined as below −6 (i.e., 6 Hz below the IAF), theta from −6 to below −2, alpha from −2 to below 2, low beta from 2 to below 10, high beta from 10 to below 20, and gamma as anything greater than or equal to 20 Hz above the IAF. However, for our analyses, data from the delta frequency band was excluded, as the EEG data collected from the delta frequency range using Emotiv headsets has been shown to have low reliability (intraclass correlation coefficient < 0.2) based on analysis of test-retest data (Prat et al., in preparation).

#### Calculating power and coherence using the IAF

**Figure 1.**
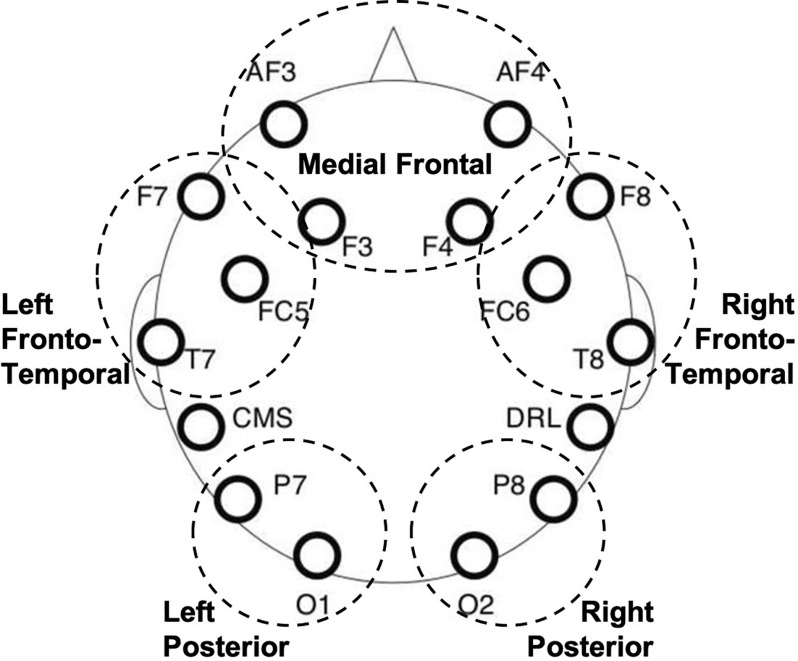
Electrode layout for the Emotiv Epoc headset, with grouped electrode regions circled and labeled. Adapted from Prat et al., 2019.

Measures of power and coherence for each frequency band were calculated using the individualized frequency bands and were averaged across electrode regions. Electrode regions were translated from previous work that has uncovered electrode clusters based on phase synchronization measuring networks of brain activity as they work together (Kepinska, Pereda, Caspers, & Schiller, 2017). Therefore, left frontotemporal electrodes included F7, FC5, and T7 and right frontotemporal electrodes included their right-hemisphere homologues; left posterior electrodes included P7 and O1, and right posterior electrodes included their right-hemisphere homologues; medial frontal electrodes included AF3, AF4, F3, and F4 (see Figure 1, adapted from Prat et al., 2019). For example, alpha power over the left frontotemporal electrodes included the average alpha power (from 2 Hz below the IAF to 2 Hz above the IAF) averaged across F7, FC5, and T7, assuming all three electrodes remained after the exclusionary steps. Coherence was first calculated as the correlated activity between every electrode pairing, at every 0.5 Hz frequency step. Then, correlated activity within each frequency band was averaged within each ROI (e.g., coherence within alpha medial frontal electrodes was calculated as the average correlated activity in the alpha range between AF3, AF4, F3, and F4) and between each ROI (e.g., coherence between medial frontal and right frontotemporal electrodes was calculated as the average correlated activity between each electrode pairing within the medial frontal electrodes and the right frontotemporal electrodes, such as AF3 to F8, AF3 to FC6, AF4 to F8, AF4 to FC6, etc.).

## RESULTS

### Analyses

To assess group differences in EEG power and coherence between the monolinguals and bilinguals, Fisher-Pitman tests were used to conduct individual *t* test comparisons between groups for (1) each frequency range within each electrode region to examine differences in power, and (2) for each frequency range within each pairing between electrode regions to examine differences in coherence. Permutation tests were used to conduct analyses because they do not assume a normal distribution, but instead calculate probabilities given the distribution and stability of effects within the provided data. For the power comparisons, false discovery rate (FDR) corrections were further applied as a strict criterion by adjusting the *p* values within a given electrode region across the frequency ranges. For the coherence comparisons, FDR adjustments were made for all comparisons involving a given electrode region. Both FDR corrected and uncorrected *p* values are reported; given the number of comparisons, it is important to extrapolate patterns of results rather than rely exclusively on significant corrected or uncorrected *p* values to reduce Type I error. Given the exploratory nature of the study and the large sample size, we further resampled half our dataset 1,000 times (ensuring a proportionate balance of monolinguals and bilinguals in each resampling) and calculated the group differences. For each electrode region and frequency range, we therefore illustrated the stability and consistency of the reported permutation *t* tests through the distribution of average group differences across 1,000 alternate subsamplings. In the following sections, significant group differences are reported, as are marginal effects when they pattern along with other significant effects (not all marginal effects are reported, but see the online supporting information located at https://www.mitpressjournals.org/doi/suppl/10.1162/nol_a_00014).

To evaluate the relation between different patterns of resting state and the linguistic and cognitive variables, we conducted permutation tests of Spearman correlations between the behavioral measures and neural measures of power for each frequency band in each electrode region. For correlations, permutation tests shuffle the observed values of each measure and recalculate the magnitude of the relation after shuffling; therefore, if the observed relation is unlikely to be reproduced by chance, then the permutated *p* value will be correspondingly low, whereas if there are many combinations of the values that produce even stronger relations than the observed relation, the permutated *p* value will be high.

We considered three aspects of language experience that are interrelated but may be differentially related to features of brain activity: proficiency, past experience, and current usage. To calculate proficiency, each participant’s self-rated proficiency scores (scale: 1–10) for speaking, understanding, and reading was averaged in each language. For past experience, we used the self-reported age of acquisition for the L2. Finally, current usage was calculated by averaging each participant’s estimated proportion of use of their L2 for speaking, listening, and reading.

Correlations between the cognitive/linguistic variables and EEG patterns were examined to determine if there were any neural factors that related to the behavioral measures. However, the bilinguals and monolinguals differed significantly in terms of both language experience and measures of cognitive control. Furthermore, bilingualism has previously been found to modulate the deployment of cognitive control, often showing correlations between measures of cognitive control and language experience where no relation exists for monolinguals (e.g., Bice & Kroll, 2015). Although language experience exists on a continuum, active bilingualism can have global modulating effects on brain activity. Such patterns suggest that certain relations may not be captured when collapsing across bilinguals and monolinguals. Therefore, in addition to correlations collapsing across groups, separate correlations were run for the bilinguals and monolinguals when examining the linguistic and cognitive variables.

### Group Differences in Power

**Figure 2.**
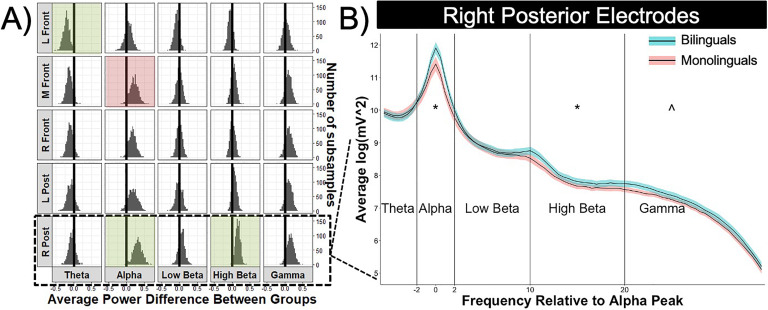
A) After resampling 50% of our dataset 1,000 times, the group differences for each electrode region and frequency range were calculated and the distribution of group difference values were plotted in the histograms. The color-shaded boxes correspond to the significant (green) or marginally significant (red) false discovery rate-corrected group differences. B) Averaged spectra in right posterior electrode region (i.e., averaged over O2 and P8) for bilinguals (blue) and monolinguals (red). Color-shaded region represents a 95% confidence interval around the averaged spectra line for each group. Individualized frequency bands with respect to the individual alpha frequency are indicated on the x-axis. Significant (*) or marginal (^) group differences are marked for relevant frequency bands.

The results of the permutation *t* tests revealed that bilinguals had significantly higher alpha power than monolinguals in right posterior electrodes, *t*(188) = 2.77, uncorrected *p* < 0.01, FDR-corrected *p* < 0.01, and marginally higher alpha power in left posterior, *t*(188) = 1.78, uncorrected *p* = 0.08, FDR-corrected *p* = 0.16, and medial frontal electrodes, *t*(188) = 1.94, uncorrected *p* = 0.05, FDR-corrected *p* = 0.08. Bilinguals also had significantly higher power in right posterior electrodes in the high beta band, *t*(188) = 2.44, uncorrected *p* = 0.01, FDR-corrected *p* = 0.02, and marginally in the gamma band, *t*(188) = 1.74, uncorrected *p* = 0.08, FDR-corrected *p* = 0.11. Monolinguals, in contrast, had significantly higher theta power in left frontotemporal electrodes than bilinguals, *t*(188) = 0.54, uncorrected *p* = 0.01; FDR-corrected *p* = 0.02. The permutated distribution of power differences for 1,000 subsamplings of the data can be found in Figure 2 in addition to the full EEG power spectrum for each group in the right posterior electrode region where the majority of differences were found.

The observed differences in power between groups could be due to larger networks of neurons firing at a frequency or to a smaller more local network with particularly high synchrony. The next analyses examined coherence differences between groups to examine whether the same frequency ranges and electrode regions would show greater coherence, which would imply that larger, more coordinated networks produced the power differences between groups.

### Group Differences in Intrinsic Coherence

Very little work has previously examined group-level differences in resting-state oscillatory dynamics between bilinguals and monolinguals. The only study to our knowledge that has reported such data revealed greater coherence in the theta and beta frequency ranges in bilinguals, particularly in posterior regions (de Frutos-Lucas et al., 2019). A mixed-effects ANOVA with group as a between-subjects variable and electrode region as a within-subjects variable on the overall coherence differences between groups revealed that bilinguals had greater coherence *between* electrode regions than monolinguals, *F*(1, 188) = 4.78, *p* = 0.03, but the two groups did not differ in their coherence *within* electrode regions, *F*(1, 188) = 0.43, *p* = 0.51. That said, all reported coherence results show greater coherence for bilinguals than monolinguals, with the exception that monolinguals had marginally higher theta coherence within the medial frontal electrodes than bilinguals, *t*(188) = 1.91, *p* = 0.06.

**Figure 3.**
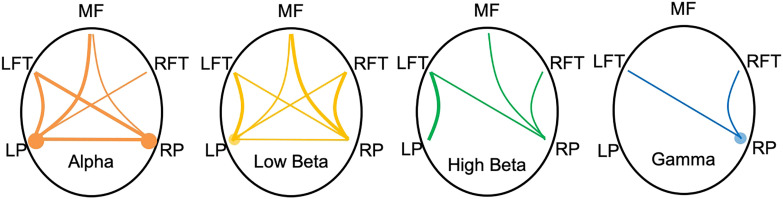
Coherence maps showing connections and frequencies in which bilinguals had greater coherence than monolinguals. Thicker lines/larger circles indicate connections between electrode regions for which bilinguals had significantly greater FDR-corrected coherence than monolinguals; thinner lines/smaller circles indicate marginally greater FDR-corrected coherence. Lines indicate connections between two electrode regions; circles indicate within-network coherence. Not depicted: monolinguals only exhibited marginally greater theta coherence than bilinguals within the medial frontal electrodes. FDR = false discovery rate.

For bilinguals, the pattern of coherence results showed that the posterior electrode regions were more connected with all other regions (see Figure 3). Bilinguals’ left posterior electrodes had significantly greater coherence with left frontotemporal electrodes in the alpha, *t*(188) = 2.94, uncorrected *p* < 0.01, FDR-corrected *p* < 0.01; low beta, *t*(188) = 2.62, uncorrected *p* < 0.01, FDR-corrected *p* = 0.02; and high beta, *t*(188) = 2.26, uncorrected *p* = 0.02; FDR-corrected *p* = 0.04, frequency ranges. Left posterior electrodes had greater coherence with the medial frontal electrodes in the alpha range, *t*(188) = 2.66, uncorrected *p* < 0.01, FDR-corrected *p* = 0.02. Left posterior electrodes had marginally greater coherence with the right frontotemporal electrodes in the alpha frequency range, *t*(188) = 2.19, uncorrected *p* = 0.03, FDR-corrected *p* = 0.06, and low beta frequency range, *t*(188) = 2.11, uncorrected *p* = 0.04, FDR-corrected *p* = 0.07. Bilinguals’ right posterior electrodes had greater coherence with the right frontotemporal electrodes in the low beta range, *t*(188) = 2.36, uncorrected *p* = 0.02, FDR-corrected *p* = 0.03, and marginally greater coherence in high beta range, *t*(188) = 2.00, uncorrected *p* < 0.05, FDR-corrected *p* = 0.07, and gamma range, *t*(188) = 2.07, uncorrected *p* = 0.04, FDR-corrected *p* = 0.06. Right posterior electrodes had greater coherence with the medial frontal electrodes in the low beta range, *t*(188) = 3.00, uncorrected *p* < 0.01, FDR-corrected *p* = 0.01, and marginally higher coherence in the alpha range, *t*(188) = 2.27, uncorrected *p* = 0.02, FDR-corrected *p* = 0.06, and high beta range, *t*(188) = 2.28, uncorrected *p* = 0.02, FDR-corrected *p* = 0.06. Right posterior electrodes had greater coherence with the left frontotemporal electrodes in the alpha range, *t*(188) = 2.69, uncorrected *p* < 0.01, FDR-corrected *p* = 0.02, and marginally higher coherence in low beta range, *t*(188) = 2.14, uncorrected *p* = 0.03, FDR-corrected *p* = 0.07; high beta range, *t*(188) = 2.01, uncorrected *p* < 0.05, FDR-corrected *p* = 0.08, and gamma range, *t*(188) = 2.09, uncorrected *p* = 0.04, FDR-corrected *p* = 0.07. Between the two posterior regions, bilinguals had significantly higher alpha coherence, *t*(188) = 3.64, uncorrected *p* < 0.001, FDR-corrected *p* = 0.001, and marginally higher low beta coherence, *t*(188) = 2.02, uncorrected *p* = 0.04, FDR-corrected *p* = 0.07. Within the left posterior electrodes, bilinguals had greater alpha coherence, *t*(188) = 3.01, uncorrected *p* = 0.01, FDR-corrected *p* < 0.01, and marginally greater low beta coherence, *t*(188) = 2.21, uncorrected *p* = 0.03, FDR-corrected *p* = 0.06. Within the right posterior electrodes, bilinguals had greater alpha coherence, *t*(188) = 3.01, uncorrected *p* < 0.01, FDR-corrected p < 0.01, and marginally greater gamma coherence, *t*(188) = 1.84, uncorrected *p* = 0.07, FDR-corrected *p* = 0.09. The full set of group comparisons of coherence data can be found in the online supporting information. The bilinguals did not have greater coherence within or between the frontal regions than the monolinguals.

The coherence results showed that bilinguals had greater alpha coherence extending from posterior regions across the head, as well as greater beta coherence extending from posterior regions across the head with stronger connections coming from the right hemisphere. While one contributing factor may be that alpha and beta are the most reliable and easiest to detect frequency ranges, it would not explain why the differences were consistently found to be stronger in bilinguals than monolinguals. The analyses of the power differences between groups revealed greater alpha power in posterior electrode regions and greater high beta power in right posterior electrodes. Combined with the coherence results, the group differences in power therefore seem to reflect that bilinguals have larger networks of neurons firing at the alpha and beta frequencies at rest compared with monolinguals, thus producing the pattern of power and coherence differences observed here. In contrast, the monolinguals only had marginally stronger coherence within the electrodes in the medial frontal region for the theta frequency, whereas they exhibited greater theta power over left frontotemporal electrodes. Those results would instead suggest that the monolinguals had slightly greater local synchrony at the theta frequency.

### Alpha

#### Peak parameters

Because individualized frequency ranges were used, one question that can be addressed specifically for alpha but not the other frequency ranges was whether the two groups differed in various alpha peak parameters: the frequency of the alpha peak (IAF), the likelihood of a peak’s presence at any given electrode, and the power of alpha at the peak frequency. Given that alpha was predicted to differ between bilinguals and monolinguals, which was confirmed in the power and coherence analyses, the expectation was that the two groups may also differ in other alpha features.

To address the question of whether one group was more or less likely to exhibit a peak in the alpha range, a logistic regression was conducted using the presence or absence of an alpha peak across each channel as the outcome variable and group and electrode as predictor variables. The logistic regression revealed that bilinguals were more likely to have an alpha peak across channels than monolinguals (β = 0.96, *SE* = 0.14, *p* < 0.001) when controlling for electrode location. To examine the peak power at the IAF in each electrode region (i.e., maximum power reached, marked by the IAF, *not* mean alpha power across frequency band), Fisher-Pitman permutation tests were used to compare monolinguals and bilinguals, which revealed that bilinguals had significantly higher peak power at the IAF than monolinguals in left posterior electrodes, *t*(188) = 2.39, *p* = 0.02; right posterior electrodes, *t*(188) = 3.47, *p* < 0.01, medial frontal electrodes, *t*(188) = 2.94, *p* < 0.01; right frontotemporal electrodes, *t*(188) = 2.33, *p* = 0.02; and marginally higher peak power in the left frontotemporal electrodes, *t*(188) = 1.69, *p* = 0.09. Finally, to examine whether the bilinguals (*M* = 10.71 Hz) and monolinguals (*M* = 10.74 Hz) differed in their IAF, we conducted a *t* test, which found no difference in IAF values between the two groups, *t*(188) = 0.2, *p* = 0.84.

Bilinguals were more likely to exhibit an alpha peak and had higher power at the observed peak than monolinguals, across the head. Given the power and coherence differences, these results further contribute to the overall pattern. That is, bilinguals have large-scale alpha synchrony at rest. Because such large networks fire in sync, bilinguals’ alpha activity must be carefully coordinated. Higher synchrony leads to higher power, especially at the specific frequency (the peak alpha frequency), which is why the peak power analyses showed differences between groups across electrode regions, whereas the averaged alpha power analyses only showed differences in (right) posterior electrodes. The higher synchrony also helps explain why the bilinguals were more likely to exhibit an alpha peak when controlling for electrode location; the greater coherence across electrodes pushes alpha through all the connections, including further forward to anterior electrodes. Alpha is predominantly found over posterior regions in general, so the whole-head coherence enables it to travel forward and produce alpha peaks more reliably in frontal electrode regions. The similar IAF values across groups is not unexpected; the two groups were of similar ages (and age is a known factor related to the frequency of the alpha peak; Klimesch, 1997), and the cognitive processes previously related to variability in the IAF, such as processing speed and intelligence, were not predicted to differ between the bilinguals and monolinguals.

#### Individual differences in language control

The remaining analyses were dedicated to investigating the underlying mechanism(s) driving the observed group differences, using measures of language experience and cognitive control. The prediction was that alpha would be related to aspects of bilingual language use that require engagement of cognitive control. Previous research has shown that bilinguals whose two languages are more balanced in proficiency exhibit greater bidirectional influence of the non-target language (for a review, see van Hell & Tanner, 2012). Because bilinguals engage inhibitory mechanisms to manage this interference (e.g., Blumenfeld & Marian, 2013; Yamasaki & Prat, 2020), past work has also shown that higher L2 proficiency, earlier age of L2 acquisition, and more balanced language use is associated with greater cognitive control among bilinguals (Kousaie et al., 2017; Luk, De Sa, & Bialystok, 2011; Yamasaki et al., 2018). Therefore, measures of language experience that capture how much bilinguals may need to engage those inhibitory control mechanisms, such as proficiency level(s), age of acquisition, and proportion of use, should be related to alpha inhibitory mechanisms.

**Figure 4.**
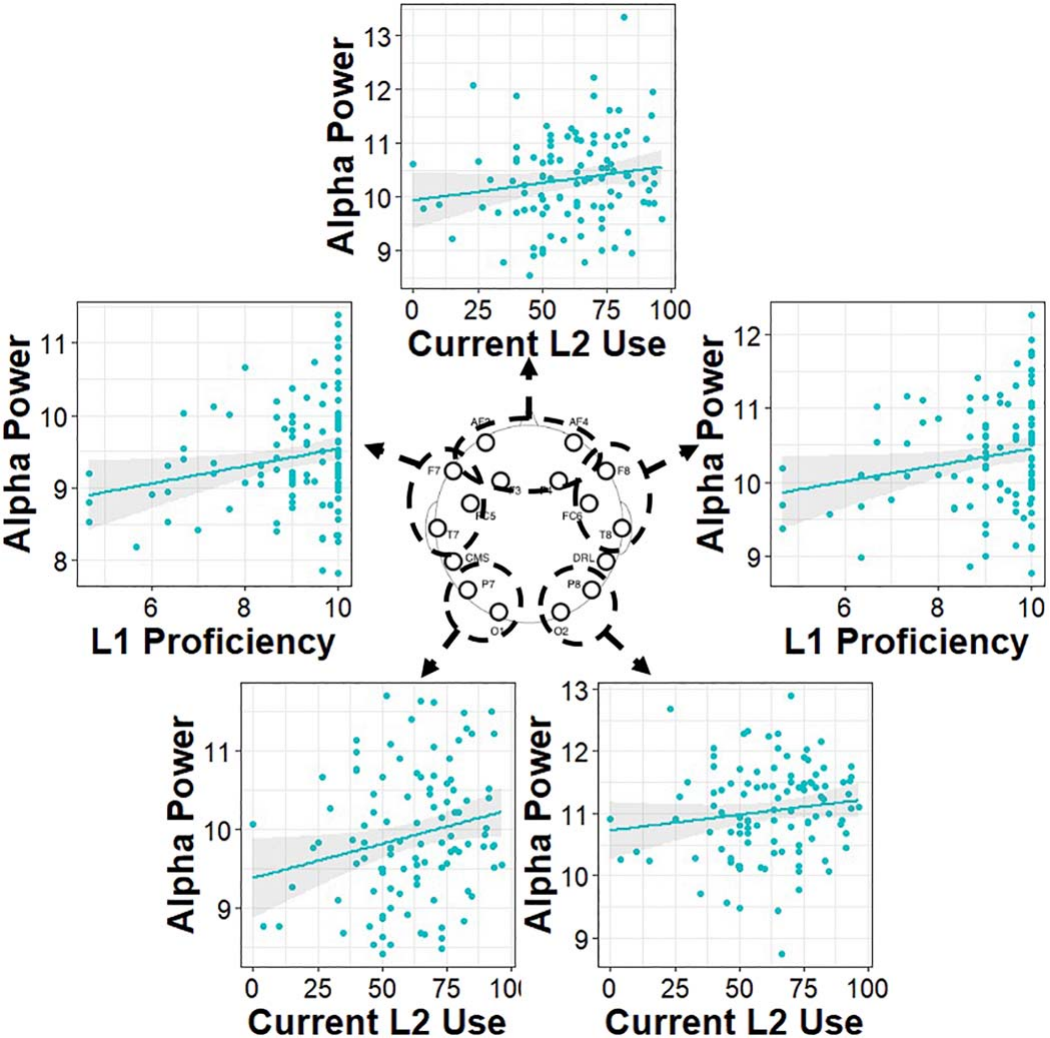
Correlations between alpha power and measures of language experience for bilingual individuals. L1 = native language; L2 = second language. Scatterplots are placed in the electrode region where the relations were observed.

**Figure 5.**
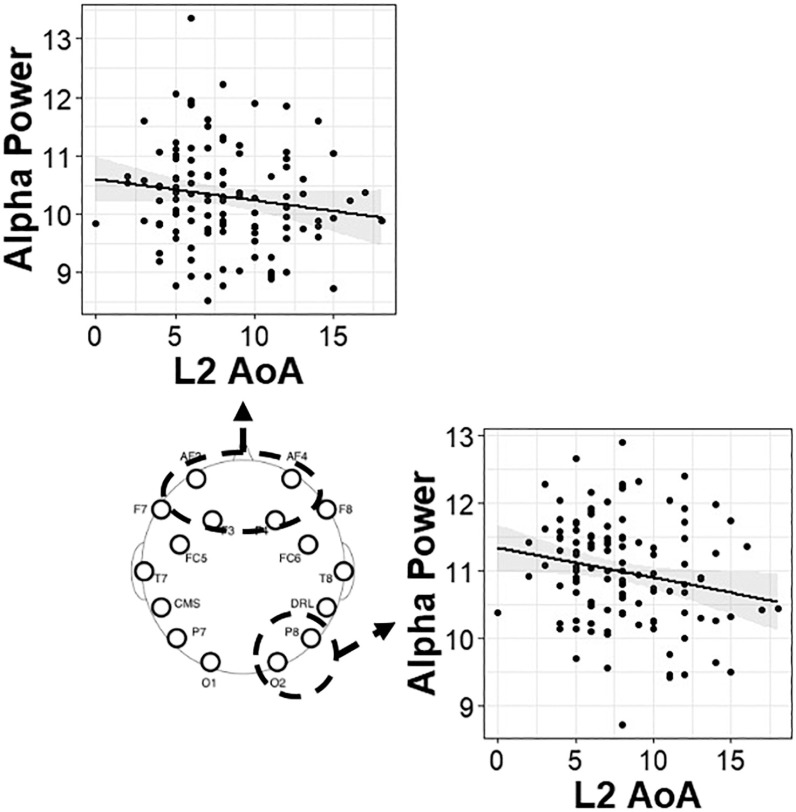
Correlations between alpha power and measures of language experience for all individuals (bilingual and monolingual). L2 AoA refers to the age of acquisition of the second-acquired language. Scatterplots are placed in the electrode region where the relation was observed.

Permutated Spearman’s correlations were conducted between alpha power in each electrode region and measures of proficiency (in the L1 and L2), past experience (L2 age of acquisition), and current usage (L2). Higher alpha power was related to earlier age of L2 acquisition, greater current L2 use, and higher L1 proficiency. Specifically, higher alpha power was related to an earlier age of acquisition of the L2, in bilinguals and collapsing across groups, in right posterior electrodes (all: *rho* = −0.20, *p* = 0.02; bilinguals: *rho* = −0.17, *p* = 0.08) and medial frontal electrodes (all: *rho* = −0.17, *p* = 0.06; bilinguals: *rho* = −0.17, *p* = 0.09). Higher alpha power was also related to a higher proportion of current L2 use in bilinguals in left posterior electrodes (*rho* = 0.20, *p* = 0.04), right posterior electrodes (*rho* = 0.16, *p* = 0.098), and medial frontal electrodes (*rho* = 0.16, *p* = 0.097). Finally, higher alpha power was also related to higher self-rated L1 proficiency for the bilinguals only in left frontotemporal electrodes (*rho* = 0.19, *p* = 0.05) and right frontotemporal electrodes (*rho* = 0.18, *p* = 0.06). Scatterplots of the relations and their spatial profiles for bilinguals can be found in Figure 4, and the scatterplots including all individuals can be found in Figure 5.

The correlations revealed that bilinguals who maintained higher proficiency in their L1 and who used their L2 more frequently had higher alpha power. Bilinguals and monolinguals who learned a second language earlier in life also had higher alpha power. The results here extend the growing body of literature in showing that alpha mechanisms support language control in bilinguals, such that the repeated and prolonged experience of managing two languages shapes alpha activity even in the absence of a specific language task.

#### Individual differences in cognitive control

Alpha is consistently found to be related to performance on cognitive control tasks when measured over (medial) frontal electrodes, but the findings are primarily drawn from alpha measured during on-task performance (e.g., Cooper et al., 2003; Klimesch et al., 2007; Sadaghiani et al., 2012). With respect to bilingualism, although it is generally accepted that bilinguals experience coactivation of their languages and recruit other cognitive mechanisms to control interference, controversy still surrounds the question of the specificity of the control that is recruited and whether the bilinguals’ practice with engaging cognitive control for cross-language conflict extends to performance on tasks that do not require language processing or control (e.g., Bialystok, Kroll, Green, MacWhinney, & Craik, 2015; Valian, 2015).

Before computing the relations between the EEG measures and Simon performance, behavioral performance was compared across groups. A mixed-effects ANOVA on the response times in the Simon task was conducted. The ANOVA on response times included group as a between-subjects variable and condition (congruent, incongruent) as a within-subjects variable, and revealed a main effect of condition, *F*(1, 192) = 524.38, *p* < 0.001, as well as a significant Group x Condition interaction, *F*(1, 192) = 7.80, *p* < 0.01. The interaction revealed that bilinguals had a larger difference in response times between congruent and incongruent trials (i.e., a larger Simon effect; *M* = 79.4 ms) than monolinguals (*M* = 61.99 ms).

A mixed-effects ANOVA was also conducted on the accuracy rates in the Simon task with group as a between-subjects variable and condition (congruent, incongruent) as a within-subjects variable. This analysis also revealed a significant main effect of condition, *F*(1, 192) = 7.23, *p* < 0.01, and a Group x Condition interaction, *F*(1, 192) = 18.94, *p* < 0.001. In contrast to the response time results, the interaction in the accuracy rates revealed that monolinguals had a larger difference in accuracy rates between the incongruent and congruent conditions (*M* = 6.98%) than bilinguals (*M* = 1.34%). In fact, among the bilinguals, there was not a significant difference in accuracy rates for the congruent and incongruent condition, *t*(102) = 0.94, *p* = 0.35.

Behaviorally, the monolinguals had a smaller response time difference between congruent and incongruent trials than bilinguals, and bilinguals had a smaller difference in accuracy rates than monolinguals. In order to capture performance on the Simon task in a single value for further correlational analyses with resting-state measures, we combined the response time and accuracy rates using the inverse efficiency score (IES; Townsend & Ashby, 1978; Vandierendonck, 2017). The IES is calculated by dividing a participant’s average response time (for correct trials only) by their accuracy rate for a given condition. We calculated the IES for congruent and incongruent trials for all participants, and then used the difference between the IES scores (incongruent–congruent) as a measure of Simon performance for further analyses. Note that larger IES scores represent slower and less accurate responses; therefore, larger Simon IES differences indicate larger costs associated with inhibitory control. Outliers were defined as any Simon score that was above or below 2.5 standard deviations of the average Simon score and were removed from analyses involving Simon performance (1 monolingual and 4 bilinguals). A *t* test on the IES values revealed that when accounting for both response times and accuracy rates, bilinguals (*M* = 66.02, *SD* = 78.09) had a smaller Simon effect than monolinguals, *t*(179) = 3.29, *p* < 0.001, *M* = 105.90, *SD* = 85.00.

**Figure 6.**
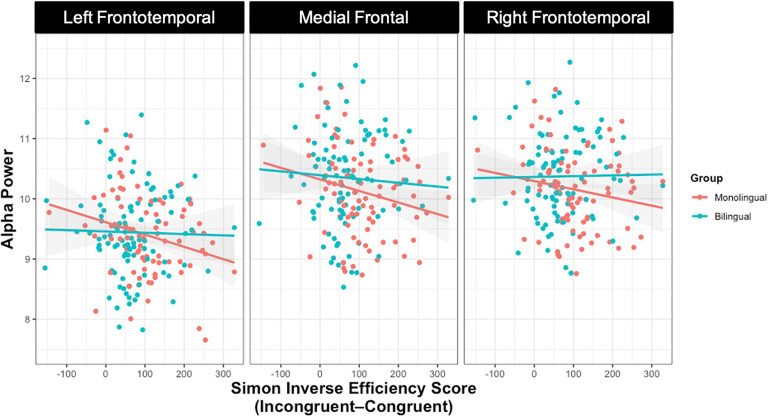
Relation between Simon performance and alpha power over three frontal electrode regions. Red line represents linear relation between Simon performance and alpha power for monolinguals and blue line represents bilinguals. Shaded region indicates 95% confidence interval surrounding the line at any given point.

A clear pattern emerged for the relation between Simon performance and EEG measures. For monolinguals only, better Simon performance (i.e., smaller difference values) was related to higher alpha power across frontal electrode regions (see Figure 6). Alpha power over left frontotemporal electrodes (*rho* = −0.25, uncorrected *p* = 0.02), medial frontal electrodes (*rho* = −0.22, uncorrected *p* = 0.04), and right frontotemporal electrodes (*rho* = −0.19, uncorrected *p* = 0.09) was negatively related to the Simon scores. For bilinguals, there were no significant relations between alpha power at any electrode region and Simon performance (all: *p* > 0.40). A visual inspection of the relation for each group shows that the lack of relation in bilinguals may be because the bilinguals have higher alpha overall (see Figure 6). As has already been shown, bilinguals had marginally higher alpha power over medial frontal electrodes than monolinguals, and the alpha power there was modulated by bilinguals’ current L2 use and age of L2 acquisition; likewise, their alpha power over bilateral frontotemporal electrodes was modulated by their L1 proficiency. Therefore, the bilinguals’ alpha power at rest may be relatively insensitive to additional variability in more general cognitive control ability because it is regularly engaged for linguistic control. Monolinguals, in contrast, revealed the expected effect whereby greater alpha power was related to better cognitive control over the expected frontal regions. For monolinguals who may not need to regularly engage alpha for language control, there remains greater variability in alpha power that can be explained by individual differences in cognitive control.

### Beta

Like alpha, beta is a general cognitive mechanism engaged in top-down control, particularly for control in working memory. Beta has also been more closely linked with language processing than alpha; beta increases during language comprehension for cohesive and coherent sentences that allow for and confirm predictions of upcoming words (Lewis & Bastiaansen, 2015; Lewis et al., 2015; Wang et al., 2012). Beta is also known to be prevalent in regions of the basal ganglia, which are also consistently found to be shaped by the demands of dual-language use (e.g., Buchweitz & Prat, 2013; Seo et al., 2018; Stocco & Prat, 2014). The results from the power and coherence analyses indicated that bilinguals had greater high beta power over right posterior electrodes and greater beta coherence with posterior electrode regions, particularly with right posterior regions. The slight tendency toward stronger effects in the right hemisphere support previous studies that have found that right-hemisphere beta activity is related to how quickly an individual learns a new language (Prat et al., 2016; Prat et al., 2019).

#### Individual differences in language experience

The relation between beta power (low and high beta) in each electrode region and the language experience measures (proficiency in each language, age of L2 acquisition, and current language usage) was examined using permutated Spearman’s correlations. Higher power in the low beta frequency range was related to higher proficiency in the L1 in the left frontotemporal electrodes for everyone (*rho* = 0.18, *p* = 0.02), as well as marginally in the right frontotemporal electrodes for the bilinguals (*rho* = 0.18, *p* = 0.07). No other significant or marginally significant relations were found.

In line with beta’s role in maintaining the “status quo,” the results suggest that beta is engaged in maintenance of the L1. The finding that both groups showed the relation in the left hemisphere electrodes but only bilinguals showed the relation in the right hemisphere electrodes is consistent with the slightly right-lateralized coherence results.

### Theta

Although not an a priori frequency of interest, the results of the group differences in power revealed that monolinguals had significantly higher theta power over left frontotemporal electrodes than bilinguals and had marginally higher theta coherence within the medial frontal electrodes. Theta is closely associated with learning and memory (e.g., Klimesch, Doppelmayr, Schimke, & Ripper, 1997), with the hippocampus as one of the known primary generators of the theta rhythm (Buzsáki, 2002; Lega, Jacobs, & Kahana, 2012). Because of its opposite relation with alpha power, typically lower theta at rest and higher theta on task are related to better performance. However, of potential relevance for the current study, it has also been related to cognitive control and activity in the anterior cingulate and prefrontal cortices when found over medial-frontal electrodes (Ishii et al., 1999; Womelsdorf, Johnston, Vinck, & Everling, 2010). Therefore, permutated Spearman’s correlations were conducted between theta power across electrode regions and the language and cognitive control measures to try to understand the mechanism driving the theta power differences.

Higher theta power over left frontotemporal electrodes was significantly related to L1 proficiency when collapsing across groups (all: *rho* = 0.20, *p* < 0.01; bilinguals only: *rho* = 0.18, *p* = 0.06). Proficiency in the L1 also differed across the two groups, *t*(126.49) = 6.13, *p* < 0.001, such that monolinguals (*M* = 9.85, *SD* = 0.40) self-rated their L1 proficiency as higher than bilinguals (*M* = 9.01, *SD* = 1.32). Therefore, higher theta power at rest could be related to L1 access and memory during periods of rest.

## DISCUSSION

The current study was among the first to examine how bilingual language use shapes the temporal dynamics of intrinsic brain activity, using data obtained from task-free EEG metrics. Active bilingual language use has been shown to shape language processing as well as more general cognitive mechanisms. Because bilinguals hold two entire languages in mind and must control interference from their nontarget language, they engage brain regions and mechanisms involved in domain-general cognitive control to suppress conflict. With repeated use and experience, the engagement of those brain regions leads to the structural and functional changes reported in fMRI studies on bilinguals and, we hypothesized, would also be present in brain activity measured via EEGs during a task-free state.

Based on previous research from at-rest and on-task EEG studies, we expected alpha and beta frequency ranges to be the primary aspects of brain activity to be affected by bilingual language experience. The prediction that alpha would be involved was strongly confirmed, with greater alpha power and coherence in bilinguals, and patterns of correlations showing that higher alpha power was related to more L2 use, earlier age of L2 acquisition, and higher L1 proficiency. The predictions about beta were also supported; bilinguals had greater high beta power in right posterior electrodes and had greater (high and low) beta coherence than monolinguals. Beta was also related to proficiency in the L1, which was found for both groups in left frontotemporal electrodes but only bilinguals in right frontotemporal electrodes. Finally, although theta was not a frequency range that was a focus, the results did reveal a number of findings for the theta range, with higher left frontotemporal theta power in monolinguals, marginally greater theta coherence within medial frontal electrodes for monolinguals, and a positive relation between theta power and L1 proficiency collapsing across groups.

### Alpha Rhythms Support Bilingual Language Control

Alpha activity was of particular interest in the current study given past findings of its role in cognitive control generally, and inhibitory processes more specifically (e.g., Klimesch et al., 2007). Alpha is the predominant rhythm found in task-free EEG measures and is one of the most studied frequency bands in EEG research. All cortical regions have been shown to generate alpha activity, as it seems to be a general mechanism for inhibiting interference from sensory inputs as well as from other neural regions (Klimesch et al., 2007). It has been related to individual differences in intelligence, memory retrieval, working memory, and many other cognitive constructs (e.g., Anokhin & Vogel, 1996; Doppelmayr et al., 2002; Doppelmayr, Klimesch, Hödlmoser, Sauseng, & Gruber, 2005; Doppelmayr et al., 2005), consistent with the fact that efficient inhibitory control is critical for widespread cognitive functioning. Coupled with the increasing body of research that suggests bilinguals rely on domain-general cognitive control mechanisms to manage interference between their coactivated languages (e.g., Abutalebi & Green, 2007; Green & Abutalebi, 2013), it was suspected that alpha may be a frequency range that would reveal differences as a function of bilingual experience.

The results of the current study, to the best of our knowledge, are the first to reveal widespread differences in alpha between bilinguals and monolinguals. As expected, bilinguals had higher alpha power and coherence than monolinguals. This is consistent with the hypothesis that using and controlling two languages requires repeated and prolonged engagement of inhibitory control, which likely relies on alpha mechanisms. Consistent with research using structural and fMRI indices, our findings suggest that repeated engagement of alpha inhibitory control mechanisms eventually shapes a distributed network of brain regions that work together to accomplish bilingual language control, as captured by the power and coherence measures in the current study.

Further evidence of this comes from the correlations showing that higher alpha power was found among bilinguals who maintained higher proficiency in their L1, used their L2 more often, and learned their L2 earlier in life. Alpha power correlated with these language experience measures primarily over posterior electrodes. While the location of underlying brain activity cannot be inferred or localized, past studies have identified activity in the parietal cortex of bilinguals that was related to global language inhibition (Guo et al., 2011). Even if the source of the brain activity cannot be specified, it is worth noting that the relations between the L2 experience measures (L2 use and L2 AoA) with alpha activity appeared in the same electrode regions where the largest group differences in alpha activity were found: right posterior, medial frontal, and left posterior electrodes. The pattern of results generally suggests that bilinguals who experienced greater cross-language competition had higher alpha power at rest.

Interestingly, while L1 proficiency was related to alpha power, L2 proficiency was not. Greater language competition is traditionally related to higher L2 proficiency under the assumption that L1 proficiency is uniformly high, so any differences in the balance of proficiency across languages is typically driven by differences in L2 proficiency. However, it is worth noting that the vast majority (102/106) of the bilinguals in this experiment were L2 English speakers living in a country where the predominant language spoken is English and where their college-level coursework is in their L2. Hence, differences in the frequency with which they use their L1 (and hence maintain proficiency in it) may drive differences in the demands they experience while using their L2.

These results are consistent with a growing body of work focusing on the role of the L1 inhibition in bilingual language use (Costa & Santesteban, 2004; Kroll, Bobb, Misra, & Guo, 2008; Marian, Bartolotti, Rochanavibhata, Bradley, & Hernandez, 2017; Meuter & Allport, 1999; Misra, Guo, Bobb, & Kroll, 2012; Peeters & Dijkstra, 2018). Various studies have demonstrated that bilinguals need to exert a greater degree of inhibitory control on their L1 to achieve L2 use. This has been observed in language-switching paradigms that reveal a larger cost in unbalanced bilinguals for switching back to the L1 after using the L2 (Meuter & Allport, 1999), or an overall slowing of the L1 in balanced bilinguals such that the L1 naming latencies are globally slower than in the L2 (Costa & Santesteban, 2004). In blocked naming paradigms, a similar phenomenon is observed; naming a set of pictures in the L1 after having named the same pictures in the L2 produces electrophysiological effects often associated with inhibitory control (Misra et al., 2012) or patterns of brain activity over the parietal cortex associated with global language inhibition (Guo et al., 2011). The interpretation of these findings has been that when a bilingual uses the L2, the L1 must be more strongly inhibited, and that when they return to using the L1, the high levels of inhibitory control imposed on the L1 must be disengaged. Extending this logic to the results of the current study, we observed higher alpha power among the bilinguals whose higher L1 proficiency in a predominantly L2 context likely required greater L1 inhibition during on-task language use. Similarly, higher alpha power was observed in bilinguals whose L1 and L2 are highly competitive due to early developmental experience in forming connections within and between languages (i.e., early L2 age of acquisition), and bilinguals who use their L2 frequently and must therefore regularly inhibit the L1 (i.e., greater current L2 use).

An alternative, yet related, interpretation of the correlational results could be that higher alpha power helps to separate and maintain the integrity of each language. For example, one study by Lev-Ari and Peperkamp (2013) showed that the L1 of language learners with low inhibitory control ability was more permeable to the influence of the L2. Other studies have similarly shown larger cross-language influence among bilinguals with lower inhibitory control (Mercier, Pivneva, & Titone, 2014; Pivneva, Mercier, & Titone, 2014; Yamasaki & Prat, 2020). These findings could lead to the interpretation that alpha serves to protect the L1 from interference and decay while these bilinguals were immersed in a primarily L2-speaking context and many had become dominant in the L2. Both interpretations of the correlation results support the hypothesis that alpha power is impacted by language experiences that create or require inhibitory control to manage cross-language interference. Importantly, while past research has reported on-task effects of language control, these findings extend our understanding by demonstrating that language control can impact how brain networks communicate even when no language task is required.

The pattern of results from the coherence and alpha parameters suggests that the bilinguals also exhibit tighter alpha tuning than do the monolinguals. The bilinguals studied had higher peak alpha power across electrode regions and were more likely to exhibit an alpha peak than were the monolinguals when controlling for electrode location. Greater alpha precision (i.e., more alpha synchrony at the specific IAF) would explain why more alpha peaks were detected in the bilinguals and why bilinguals had higher peak power at the IAF. More jittered alpha synchrony, as in the monolinguals, would result in less pronounced peaks in the alpha band, thus reducing the number of alpha peaks detected and producing lower power at the IAF. When the jittered alpha power spread out across the frequency band was averaged, the group differences in the averaged alpha frequency would thus be reduced, as found in the pattern of results reported here.

Greater neural precision in bilinguals has been found in other studies examining the sound frequency domain. Krizman, Marian, Shook, Skoe, and Kraus (2012) compared the auditory brainstem response, or the neural encoding of the fundamental frequency of an incoming auditory signal, between bilinguals and monolinguals. They found that bilinguals exhibited more precise neural encoding of incoming speech sounds. Furthermore, the bilinguals’ neural responses to speech sounds embedded in noise were related to measures of cognitive control, whereas the monolinguals’ neural responses were not. Given the greater variability in bilingual language experience, bilinguals may be required to develop more precise representations to successfully extract signal from their noisier environments. Other studies have directly linked alpha oscillations to selective listening in noisy contexts (Strauß et al., 2014). The results from the current study, showing greater alpha precision in bilinguals, seem to suggest that alpha mechanisms used for cognitive control may be more precisely tuned in bilinguals to help extract signal from the noisier environments they encounter, which likely extend beyond the domain of auditory processing to other cognitive domains.

An alternative interpretation of the pattern of results in the current study is that the higher peak alpha power observed in bilinguals may have been driven by the fact that bilinguals were more likely to have an alpha peak. However, if that were the case, then removing electrodes without a peak should have resulted in similar peak alpha power for the two groups. An analysis of only electrodes with detected peaks showed the exact same pattern as the analyses reported here that included electrodes without peaks. In general, relatively little is known about individual differences in alpha peaks, including their presence or absence, but the results of the current study seem to suggest that bilingual language experience increases alpha precision as well as alpha power and coordination (coherence).

In addition to the group differences in alpha, we expected that alpha activity would be related to measures of cognitive control, measured via the Simon task. The correlational analyses did reveal a significant relation between frontally distributed alpha activity and cognitive control, but for monolinguals only. Visually, it seemed that bilinguals’ alpha was unrelated to individual differences in Simon performance due to their overall higher alpha activity. Bilinguals’ alpha activity is so intertwined with their language control needs, which are relatively constant, that their intrinsic alpha activity is primarily shaped by individual differences in language experience. In contrast, monolinguals do not engage alpha for such frequent daily experiences as dual-language control, and therefore their alpha activity at rest is more closely related to how often or how well they engage domain-general cognitive control mechanisms.

### Beta Rhythms Help the Native Language

As expected based on findings from the de Frutos-Lucas et al. (2019) study, our results showed greater beta coherence in the bilingual speakers, and extended the findings to show greater beta power in bilinguals as well. Similar to the de Frutos-Lucas study and to the alpha findings in the current study, higher beta coherence was centralized in posterior electrodes, particularly within the right-hemisphere electrodes. Further evidence from the correlational analyses revealed that bilateral frontal low beta power correlated with the bilinguals’ L1 proficiency, whereas the monolinguals’ L1 proficiency was related only to left frontal beta. Together, this pattern of results supported our hypothesis that bilingual language use would impact beta activity.

The beta results were slightly skewed toward the right hemisphere, in line with other studies that have found greater right hemisphere involvement in bilingualism and L2 learning (Kepinska et al., 2017; Mamiya, Richards, Coe, Eichler, & Kuhl, 2016; Prat et al., 2016; Qi, Han, Garel, San Chen, & Gabrieli, 2015). The right hemisphere is recruited to help with the processing of subordinate word meanings (Wlotko, Lee, & Federmeier, 2010), metaphors (Yang, 2014), and the less proficient of a bilingual’s two languages (Chee, Hon, Lee, & Soon, 2001; Leonard et al., 2011), and to integrate different timescales of language information (Giraud & Poeppel, 2012; Hickok & Poeppel, 2007). Many roles of the right-hemisphere therefore seem to lend support for difficult or unexpected linguistic phenomena (Dräger et al., 2004; Rapp, Leube, Erb, Grodd, Kircher, 2004; van Ettinger-Veenstra et al., 2010), congruent with the idea that bilinguals experience greater demands from dual-language use.

An increasing number of studies have related language learning success to right-hemisphere structure and function. For example, stronger white matter integrity in the right hemisphere has repeatedly been related to more facile L2 learning (Mamiya et al., 2016; Qi et al., 2015). Of closer relevance to the current experiment, increased beta activity over right-hemisphere electrodes measured at rest has been shown to predict the rate of learning natural languages in adulthood (Prat et al., 2016, 2019) and, more recently, computer programming languages (Prat, Madhyastha, Mottarella, & Kuo, 2020). The results of the current study, which show that bilinguals have greater beta power and coherence over right-hemisphere electrodes, may provide an important link between research on individual differences in right-hemisphere beta in L2 learning and research showing that bilinguals are better at acquiring new languages than monolinguals (e.g., Bogulski et al., 2018; Kaushanskaya & Marian, 2009). The results here showed that bilinguals have greater right-hemisphere beta coherence than monolinguals, providing one possible mechanism that enables better and faster learning of a new language.

Overall, beta frequencies appear to be heavily implicated in dual-language success. The observation that beta power was related to L1 proficiency for both groups in the left hemisphere electrodes suggests it plays a role in language maintenance and fluency; the added right-hemisphere contribution in bilinguals may speak to the greater demands on bilinguals due to dual-language use.

### Coherence as Coordination

Some of the most striking results of the current study came from the coherence analyses. Bilinguals had significantly higher alpha and beta coherence between posterior electrodes and almost every other electrode region (see Figure 3). Low and high beta coherence was especially stronger for bilinguals in right-hemisphere electrodes. Many researchers have argued and demonstrated that dual-language use requires greater domain-general coordination (e.g., Abutalebi & Green, 2007; Blanco-Elorrieta & Pylkkänen, 2016; Calabria, Costa, Green, & Abutalebi, 2018; Kroll & Bialystok, 2013). Language processes in bilinguals and L2 learners recruit more broadly distributed regions (including the right hemisphere especially for the less proficient language), and language processes are more closely coordinated with domain-general cognitive control, monitoring, and attentional mechanisms (e.g., Bice & Kroll, 2015; Teubner-Rhodes et al., 2016; Zirnstein, Van Hell, & Kroll, 2018). Previous research has identified increased activity in the right posterior parietal cortex under more difficult word-finding conditions, which researchers have associated with greater sustained attention and executive control that is drawn upon to resolve difficult linguistic processes (Dräger et al., 2004). Other recent research using fMRI has shown that the coupling between the right inferior parietal cortex with other cortical areas (cingulate cortex, precentral gyrus, superior frontal gyrus, precuneus) was modulated during on-task switching between languages (Tabassi Mofrad & Schiller, 2019), again emphasizing the role of (right) posterior coherence for language control. The coherence results from the current study suggest that the additional demands of dual-language use are due not only to greater *difficulty* but also to greater *coordination* of language and cognitive control.

These results are largely in line with the de Frutos-Lucas et al. (2019) study, as well as the wealth of other resting-state MRI literature that demonstrates greater white matter connectivity among bilinguals and language learners that has been attributed to long-term consequences of dual-language control and use (e.g., DeLuca et al., 2019; Li et al., 2014). As in the de Frutos-Lucas et al. study, the results of the current study revealed that bilinguals had much greater and broader coherence. Moreover, the hub of the coherent activity appeared primarily in posterior regions. The posterior nature of the results in both studies suggests that bilingual experience significantly shapes how posterior brain networks coordinate with other areas of the brain, which is also consistent with the bilingual anterior-to-posterior and subcortical shift (BAPSS) model (Grundy et al., 2017). One difference between the two studies was that de Frutos-Lucas et al. primarily found effects in the theta and beta range, whereas here the main differences were in alpha and beta. The discrepancy in results could be due to the use of individualized frequency ranges in the current study. Across the lifespan, a person’s IAF follows an inverted U-shaped curve, such that IAF is at its highest frequency in young and middle adulthood but slows significantly in older adults (Klimesch, 1999), which was the population used in de Frutos-Lucas et al. (2019). It is possible that their use of fixed frequency ranges may have inadvertently attributed activity to the theta range that was actually slowed alpha activity in their older adult population. Discrepancies between the results of the current study showing large-scale alpha differences and their results centralized in theta may therefore reflect methodological differences in defining the frequency ranges. Regardless of the discrepancies, the results from the current study extend the small body of previous work on oscillatory differences between bilinguals and monolinguals, and add to the growing literature demonstrating that dual-language use places demands on cognitive networks that require greater connectivity and coordination.

### Theta and Native Language Proficiency

While alpha and beta were the primary a priori frequencies of interest, a number of the results also revealed effects in theta. Theta has been implicated in learning and memory (e.g., Klimesch et al., 1997) and is widely found in the hippocampus and its associated regions (Buzsáki, 2002; Lega, Jacobs, & Kahana, 2012). Monolinguals in the current study had significantly higher L1 proficiency, in addition to higher theta power over left frontotemporal electrodes and marginally higher theta coherence within medial-frontal electrodes than bilinguals. The spatial extent of the group differences in theta was very restricted and limited to monolinguals. The correlations showed that higher theta power over left frontotemporal electrodes was related to higher proficiency in L1 for both groups. The consistency across the group differences and correlations suggests that left-frontal theta activity is related to language proficiency.

While no strong conclusion can be made about the mechanism driving the theta differences based on these data alone, it is interesting to note that left frontotemporal theta was the only frequency found to have greater power in monolinguals than bilinguals, that it was related to language performance, and that it was restricted in its spatial profile. Many have argued that one consequence of dual-language use is that the networks supporting language must be cast wider to coordinate with various other regions for control and conflict resolution; the reverse side of that argument is that monolingual language processing remains, by comparison, relatively focal. The specificity of the results here supports that argument, although a strong conclusion cannot be made with respect to the exact function of theta activity and its role in language processing.

These findings are consistent with previous studies on neural oscillations involved in online speech processing, which propose that the frequency of theta is ideal for integrating acoustic information at the level of the syllable (e.g., Giraud & Poeppel, 2012; Meyer, 2018). The models suggest that the syllable-level processing primarily takes place in the left hemisphere, whereas other oscillations are better poised to process language in parallel in the right hemisphere. Thus, the theta findings in the current study may be related to language processing, fluency, and retrieving information for successful L1 comprehension.

### Summary and Conclusion

The results of the current study provide an important first step toward a more comprehensive understanding of the impact of bilingual language experience on brain function. Given that the reported data were collected from a task-free period, the findings illuminate how bilingualism permeates brain function, even in the absence of language use. Accumulating evidence suggests that bilingual language experience and more dynamic facets of language use both have measurable impacts on the brain’s functional connectivity. Future work on the topic of task-free EEG measures should consider the interplay between neural oscillation changes observable at rest with task-based EEG and behavioral measures.

One strength and limitation of the current study was that it included a heterogeneous sample of bilingual speakers. While such a breadth of language experiences strengthens generalizability, there may be further effects of dual-language use that were not captured amid such variability. Future work could consider the role of language typology or linguistic distance between the bilinguals’ known languages to probe how features like tones or morphological complexity may modulate the observed effects. A limitation of the current study was the reliance on self-report measures of proficiency, which could be improved in future studies by including objective measures of proficiency in each language.

One particularly interesting aspect of the sample included in the current study was that the majority of bilinguals (96%) did not report English as their L1, and thus were immersed in their L2. The majority of those (64%) had reportedly switched dominance, thus self-rating their proficiency in English as higher than in their L1. The consequences of such immersion are well documented (Rahmani, Sobhani, & Aarabi, 2017; Stein, Winkler, Kaiser, & Dierks, 2014), while the consequences of a language dominance switch are virtually unknown. Some work has shown that bilinguals in an immersion context apply global inhibition to the entire L1 (Linck, Kroll, & Sunderman, 2009), and that global language inhibition can be observed in patterns of brain activity over the parietal cortex (Guo et al., 2011). Other work has found that the contexts in which bilinguals use their languages shape how their cognitive control mechanisms are engaged (e.g., Abutalebi & Green, 2007). Given that the bilinguals were immersed in English, it is perhaps unsurprising that phenomenologically, English felt more available and fluent to them at the moment. An interesting question for future research is whether alpha helps with dominance switches in the context of immersion; it may be that bilinguals who can engage alpha more readily to reduce the activity of their L1 may be more likely to switch dominance in an immersion context to the majority language. Likewise, as more research in bilingualism calls for a greater emphasis on differences in bilingual language experience as a function of how bilinguals use and interact in their two languages (e.g., Green & Abutalebi, 2013), a future investigation could take a more nuanced look at interactional contexts by soliciting detailed information regarding the context of language use for more sophisticated language experience measures, like entropy (Gullifer & Titone, 2020), to provide additional insights into the cognitive mechanisms supporting dual-language use.

In summary, the current study was among the first to examine differences in task-free brain activity, measured via EEGs, between bilingual and monolingual speakers. The results revealed several systematic differences that were related to measures of language experience and cognitive control. Alpha and beta, the frequency ranges in which the majority of the differences were found, were related to features of bilingual experiences known to affect requirements for language control and language maintenance, respectively. These findings provide further support to the growing body of research that demonstrates how brain function is shaped by significant life experiences.

## FUNDING INFORMATION

Kinsey Bice, Washington Research Foundation Innovation Postdoctoral Fellowship in Neuroengineering. Chantel S. Prat, Office of Naval Research, Award ID: N00014-17-1-2607.

## AUTHOR CONTRIBUTIONS

Kinsey Bice: Conceptualization; Methodology; Formal analysis; Writing—original draft, Writing—review & editing; Visualization. Brianna L. Yamasaki: Methodology; Investigation; Data curation; Writing—review & editing. Chantel S. Prat: Conceptualization; Resources; Writing—review & editing; Visualization; Supervision; Project administration; Funding acquisition.
