# Supplementary material for: Bilingual Language Experience Shapes Resting-State Brain Rhythms
Source: Neurobiol Lang (Camb). 2020 Jul 1;1(3):288–318. doi: 10.1162/nol_a_00014 (PMC10158654; doi:10.1162/nol_a_00014)
Supplement: Supplementary file 1 [file nol-1-3-288-s001.pdf]

Full set of correlational results for each linguistic or cognitive variaible included, using permutated Spearman's rho. Values in the tables are the rho values for the relation between the individual's score on the variable and their average power for the indicated electrode region and frequency range; italicized values were maringally significant, bolded values indicate significant relations

L1 Proficiency
Collapsed Across Groups

| | Theta | Alpha | Low Beta | High Beta | Gamma |
|---|---|---|---|---|---|
| Left Fronto-Temporal | **0.20** | 0.07 | **0.18** | 0.09 | 0.01 |
| Medial Frontal | 0.12 | -0.04 | 0.10 | 0.02 | 0.03 |
| Right Fronto-Temporal | 0.06 | 0.00 | 0.09 | 0.06 | -0.08 |
| Left Posterior | 0.06 | -0.09 | 0.05 | -0.03 | 0.05 |
| Right Posterior | 0.07 | -0.11 | 0.04 | -0.09 | -0.01 |

L1 Proficiency
Bilinguals Only

| | Theta | Alpha | Low Beta | High Beta | Gamma |
|---|---|---|---|---|---|
| Left Fronto-Temporal | *0.18* | **0.19** | **0.20** | 0.05 | 0.04 |
| Medial Frontal | 0.13 | 0.10 | 0.13 | 0.05 | 0.11 |
| Right Fronto-Temporal | 0.11 | *0.18* | *0.18* | 0.11 | -0.04 |
| Left Posterior | 0.06 | 0.01 | 0.09 | 0.01 | 0.11 |
| Right Posterior | 0.11 | 0.03 | 0.12 | 0.02 | 0.09 |

L1 Proficiency
Monolinguals Only

| | Theta | Alpha | Low Beta | High Beta | Gamma |
|---|---|---|---|---|---|
| Left Fronto-Temporal | 0.04 | -0.08 | -0.01 | -0.04 | -0.05 |
| Medial Frontal | -0.05 | -0.13 | 0.01 | -0.09 | 0.03 |
| Right Fronto-Temporal | -0.09 | -0.15 | -0.03 | -0.04 | -0.08 |
| Left Posterior | 0.01 | -0.14 | -0.04 | -0.08 | 0.11 |
| Right Posterior | -0.04 | -0.13 | -0.03 | -0.11 | -0.07 |

L1 Current Usage
Bilinguals Only

| | Theta | Alpha | Low Beta | High Beta | Gamma |
|---|---|---|---|---|---|
| Left Fronto-Temporal | -0.07 | -0.13 | -0.01 | -0.04 | -0.03 |
| Medial Frontal | -0.09 | *-0.18* | -0.10 | -0.09 | -0.08 |
| Right Fronto-Temporal | -0.04 | -0.08 | -0.01 | 0.00 | -0.05 |
| Left Posterior | *-0.17* | **-0.24** | -0.11 | -0.12 | -0.07 |
| Right Posterior | -0.05 | *-0.18* | -0.07 | -0.05 | -0.01 |

L2 Proficiency
Collapsed Across Groups

| | Theta | Alpha | Low Beta | High Beta | Gamma |
|---|---|---|---|---|---|
| Left Fronto-Temporal | -0.11 | 0.03 | -0.10 | -0.08 | -0.03 |

| | | | | | |
|---|---|---|---|---|---|
| Medial Frontal | -0.05 | 0.08 | -0.06 | -0.09 | 0.04 |
| Right Fronto-Temporal | -0.05 | 0.04 | -0.06 | -0.04 | 0.11 |
| Left Posterior | 0.03 | 0.13 | -0.04 | 0.00 | 0.09 |
| Right Posterior | 0.01 | *0.17* | -0.03 | -0.04 | 0.01 |

L2 Proficiency
Bilinguals Only

| | Theta | Alpha | Low Beta | High Beta | Gamma |
|---|---|---|---|---|---|
| Left Fronto-Temporal | 0.01 | 0.04 | -0.04 | -0.06 | -0.09 |
| Medial Frontal | 0.04 | 0.05 | -0.01 | -0.11 | -0.08 |
| Right Fronto-Temporal | 0.04 | 0.01 | -0.03 | -0.07 | 0.01 |
| Left Posterior | 0.07 | 0.13 | 0.00 | -0.03 | 0.00 |
| Right Posterior | 0.04 | 0.13 | -0.03 | -0.11 | -0.08 |

L2 Age of Acquisition
Collapsed Across Groups

| | Theta | Alpha | Low Beta | High Beta | Gamma |
|---|---|---|---|---|---|
| Left Fronto-Temporal | 0.14 | -0.11 | -0.01 | -0.02 | 0.02 |
| Medial Frontal | -0.03 | *-0.17* | -0.08 | -0.07 | -0.04 |
| Right Fronto-Temporal | 0.06 | -0.14 | -0.04 | -0.05 | -0.02 |
| Left Posterior | 0.04 | -0.07 | 0.06 | 0.03 | -0.04 |
| Right Posterior | -0.09 | **-0.20** | -0.07 | -0.06 | 0.02 |

L2 Age of Acquisition
Bilinguals Only

| | Theta | Alpha | Low Beta | High Beta | Gamma |
|---|---|---|---|---|---|
| Left Fronto-Temporal | 0.04 | -0.13 | -0.08 | -0.04 | 0.07 |
| Medial Frontal | -0.14 | *-0.17* | -0.15 | -0.09 | 0.04 |
| Right Fronto-Temporal | -0.01 | -0.13 | -0.08 | -0.05 | 0.07 |
| Left Posterior | 0.02 | -0.06 | 0.04 | 0.06 | 0.04 |
| Right Posterior | -0.14 | *-0.17* | -0.08 | -0.02 | 0.09 |

Simon Effect- Inverse Efficiency Score
Collapsed Across Groups

| | Theta | Alpha | Low Beta | High Beta | Gamma |
|---|---|---|---|---|---|
| Left Fronto-Temporal | -0.04 | -0.12 | -0.06 | -0.01 | 0.01 |
| Medial Frontal | -0.02 | -0.12 | -0.06 | -0.05 | -0.07 |
| Right Fronto-Temporal | 0.01 | -0.10 | -0.06 | -0.06 | 0.02 |
| Left Posterior | -0.11 | *-0.13* | -0.10 | -0.01 | 0.02 |
| Right Posterior | -0.02 | -0.10 | -0.08 | 0.00 | -0.04 |

Simon Effect- Inverse Efficiency Score
Bilinguals Only

| | Theta | Alpha | Low Beta | High Beta | Gamma |
|---|---|---|---|---|---|
| Left Fronto-Temporal | -0.09 | 0.00 | -0.07 | 0.01 | 0.13 |
| Medial Frontal | -0.04 | 0.03 | -0.06 | -0.03 | 0.02 |
| Right Fronto-Temporal | 0.04 | 0.08 | -0.05 | 0.00 | 0.13 |

| | | | | | |
|---|---|---|---|---|---|
| Left Posterior | *-0.17* | -0.05 | -0.12 | -0.02 | 0.04 |
| Right Posterior | 0.04 | 0.08 | -0.05 | 0.00 | 0.13 |

Simon Effect- Inverse Efficiency Score
Monolinguals Only

| | Theta | Alpha | Low Beta | High Beta | Gamma |
|---|---|---|---|---|---|
| Left Fronto-Temporal | -0.12 | **-0.25** | -0.14 | -0.11 | -0.13 |
| Medial Frontal | -0.10 | **-0.22** | -0.09 | -0.09 | -0.11 |
| Right Fronto-Temporal | -0.08 | *-0.19* | -0.06 | -0.09 | -0.02 |
| Left Posterior | -0.10 | -0.18 | -0.13 | 0.04 | 0.08 |
| Right Posterior | -0.08 | -0.10 | -0.09 | 0.03 | 0.02 |
