# Supplementary material for: Bilingual Language Experience Shapes Resting-State Brain Rhythms
Source: Neurobiol Lang (Camb). 2020 Jul 1;1(3):288–318. doi: 10.1162/nol_a_00014 (PMC10158654; doi:10.1162/nol_a_00014)
Supplement: Supplementary file 2 [file nol-1-3-288-s002.pdf]

Group differences in power (bilinguals - monolinguals) for each electrode region and frequency range. Values are the mean difference; italicized values are marginally significant after FDR-correction of the permutated t-tests, bold values indicate significant group difference after FDR-correction

	Theta	Alpha	Low Beta	High Beta	Gamma
Left Fronto-Temporal	**-0.20**	0.07	-0.05	-0.03	0.03
Medial-Frontal	-0.11	*0.23*	0.02	0.07	0.10
Right Fronto-Temporal	-0.07	0.17	0.02	0.04	0.12
Left Posterior	-0.08	0.22	0.01	0.07	0.09
Right Posterior	-0.07	**0.31**	0.06	**0.16**	0.17

Group differences in coherence (bilinguals - monolinguals) for every connection between electrode regions and for each frequency range. Values are the mean difference; italicized values are marginally significant after FDR-correction of the permutated t-tests, bold values indicate significant group difference after FDR-correction

Connection between:						
Electrode Region 1:	Electrode Region 2:	Theta	Alpha	Low Beta	High Beta	Gamma
Left Fronto-Temporal	Medial Frontal	0.005	0.008	0.004	0.006	0.006
Left Fronto-Temporal	Right Fronto-Temporal	-0.002	-0.007	0.000	0.006	0.007
Left Fronto-Temporal	Left Fronto-Temporal	0.008	0.002	0.002	0.005	0.005
Left Posterior	Left Fronto-Temporal	0.011	**0.021**	**0.014**	**0.013**	0.009
Left Posterior	Medial Frontal	0.005	**0.020**	0.009	0.007	0.004
Left Posterior	Right Fronto-Temporal	0.007	*0.012*	*0.011*	0.008	0.007
Left Posterior	Right Posterior	0.009	**0.021**	*0.012*	0.010	0.009
Left Posterior	Left Posterior	0.008	**0.024**	*0.011*	0.010	0.007
Medial Frontal	Medial Frontal	*-0.018*	-0.010	-0.014	-0.005	0.001
Right Fronto-Temporal	Medial Frontal	0.002	-0.010	0.003	0.006	0.007
Right Fronto-Temporal	Right Fronto-Temporal	-0.007	-0.012	-0.002	0.005	0.007
Right Posterior	Left Fronto-Temporal	0.009	**0.011**	*0.009*	*0.010*	*0.010*
Right Posterior	Medial Frontal	0.013	*0.012*	**0.017**	*0.013*	0.011
Right Posterior	Right Fronto-Temporal	0.006	0.009	**0.016**	*0.014*	*0.013*
Right Posterior	Right Posterior	0.005	**0.013**	0.008	0.010	*0.011*
